# Socioeconomic determinants of medicines use in Central Eastern Europe: the role of pharmaceutical policy in reducing inequalities

**DOI:** 10.1186/2052-3211-8-S1-O12

**Published:** 2015-10-05

**Authors:** Sabine Vogler, August Österle, Susanne Mayer

**Affiliations:** 1 WHO Collaborating Centre for Pricing and Reimbursement Policies, Department of Health Economics, Gesundheit Österreich GmbH (Austrian Public Health Institute), Vienna, 1010, Austria; 2 Institute for Social Policy, Department of Socioeconomics, Vienna University of Economics and Business, Vienna, 1020, Austria; 3 Department of Health Economics, Centre for Public Health, Medical University Vienna, Vienna, 1090, Austria

## Background

Even though access to essential medicines is a human right, inequalities in access resulting in differences in medicine use between socioeconomic groups are known from several countries world-wide. However, the socioeconomic determinants of medicine use in Central and Eastern European countries (CEECs) have not yet been explored. For a sample of eight countries (Bulgaria, Czech Republic, Hungary, Latvia, Poland, Romania, Slovenia, and Slovakia), this study thus aims to analyse whether socioeconomic status influences medicine use and investigates to what extent observed inequalities can be explained by current (lack of) pharmaceutical policies and how policies can help reduce existing inequalities.

## Methods

Quantitative analyses on socioeconomic determinants of medicine use (based on cross-sectional data from the first wave of the European Health Interview Survey) and qualitative analyses of the national pharmaceutical policy framework (based on information produced in the Pharmaceutical Pricing and Reimbursement Information project) for the time period 2006-2009 were conducted.

## Results

Women and people with chronic diseases and lower self-assessed health were found to have a higher likelihood to take medicines. In the field of non-prescribed medicines that were usually not reimbursed by the public payers, people with higher education and/or higher income were attributed a higher chance of consuming these medicines in seven of the surveyed countries. Regarding prescribed medicines, such a socioeconomic gradient in medicine use was only observed in three countries (Latvia, Poland, and Romania). The analysis of pharmaceutical policies identified private expenditure, overall investment in health systems, rational use of medicines and clear procedures for inclusion of medicines into reimbursement as major factors that co-determine this socioeconomic gradient in medicine use in Central and Eastern Europe. Latvia, Poland and Romania had a comparably high share of patients' contributions, and Latvia and Romania were furthermore strongly hit by the global financial crisis and reacted through cost-containment measures.

## Conclusions

A socioeconomic gradient in medicine consumption was found in the CEECs, particularly regarding non-prescribed medicines and, as a trend, it tended to favour the more affluent population. Public pharmaceutical policies usually addressed prescribed, reimbursed medicines, and in several CEECs they appeared to have positively contributed to improving access to these medicines for people with a lower socioeconomic status. Pharmaceutical policies aiming at reducing inequalities in medicine use require not only a consideration of the role of co-payments and other private expenditure but also adequate investment in medicines and transparent and clear processes regarding the inclusion of medicines into reimbursement.

